# Proteomics in Melanoma Biomarker Discovery: Great Potential, Many Obstacles

**DOI:** 10.1155/2011/181890

**Published:** 2011-10-11

**Authors:** Michael S. Sabel, Yashu Liu, David M. Lubman

**Affiliations:** Department of Surgery, University of Michigan Health Systems, 1500 East Medical Center Drive, Ann Arbor, MI 48109, USA

## Abstract

The present clinical staging of melanoma stratifies patients into heterogeneous groups, resulting in the application of aggressive therapies to large populations, diluting impact and increasing toxicity. To move to a new era of therapeutic decisions based on highly specific tumor profiling, the discovery and validation of new prognostic and predictive biomarkers in melanoma is critical. Genomic profiling, which is showing promise in other solid tumors, requires fresh tissue from a large number of primary tumors, and thus faces a unique challenge in melanoma. For this and other reasons, proteomics appears to be an ideal choice for the discovery of new melanoma biomarkers. Several approaches to proteomics have been utilized in the search for clinically relevant biomarkers, but to date the results have been relatively limited. This article will review the present work using both tissue and serum proteomics in the search for melanoma biomarkers, highlighting both the relative advantages and disadvantages of each approach. In addition, we review several of the major obstacles that need to be overcome in order to advance the field.

## 1. Introduction

The field of oncology is rapidly attempting to move to a new era of personalized therapy, where individualized therapeutic decisions are based on highly specific tumor profiling. For this to become a reality, the discovery and validation of new prognostic and predictive biomarkers are necessary. This goal seems increasingly realistic thanks to high-throughput screening methods, particularly genomic profiling. The large-scale analysis of gene expression has improved our knowledge of tumorigenesis, invasion, and metastasis. More recently, gene expression assays have helped guide therapeutic decisions, such as the use of multigene assays for decisions regarding systemic therapy in breast cancer [1, 2]. However, despite continued success in the preclinical setting, clinical translation has been slow and several tumor types present unique challenges to the use of genomic profiling.

One example is melanoma. The present staging system for melanoma, using Breslow thickness, ulceration, mitotic rate, and the presence of regional and distant metastases, stratifies patients into heterogeneous groups, with wide variability in outcome or response to therapy. Clinically, this results in applying more aggressive surgical and adjuvant therapies to large populations, diluting the impact of therapy while exposing more patients to toxicity. For those treating melanoma, better prognostic and predictive markers in melanoma are sorely needed, but to date have been elusive. The high-throughput analysis of genomic data requires fresh tissue from a large number of primary tumors. This presents a unique challenge in melanoma where the primary is often only a few millimeters in size, with no residual tissue after the diagnosis has been made. For this reason, proteomics appears to be an ideal choice for the discovery of new prognostic and predictive biomarkers in melanoma. 

There are other potential advantages to proteomics over genomics. Genes are transcribed into mRNA, but because cells can use alternative splicing, there is no one-to-one relationship between the genome and the transcript. The transcripts are further translated into proteins, which often undergo posttranslational modifications (PTMs), or can be aberrant in cancer cells. Therefore, one gene can result in several different protein isoforms. Protein structure can also be influenced by environmental factors, including interaction with other proteins, degradation, or compartmentalization of proteins within protein complexes. As the structure and availability of the final versions of the proteins ultimately determine the behavior of the cell, high-throughput screening methods for changes in protein expression may be better suited to identify biomarkers with prognostic or predictive value.

## 2. Biomarkers in Melanoma

The clinical potential for melanoma biomarkers covers the spectrum of disease. Protein changes associated with the transition from melanocyte to atypia or dysplasia and ultimately to melanoma could be used to aid in diagnosis or to screen high-risk patients. Proteins associated with pathophysiology and malignant properties could be used to further classify melanoma, stratifying patients by risk of recurrence in order to better select surgical and adjuvant treatments. Likewise, protein expression (baseline or changes in expression) may predict response to specific therapies, so that selection of systemic therapies can be tailored to the individual patient. The detection of low levels of melanoma-associated proteins in the serum may also lead to early recognition of recurrent disease or monitoring the response to therapy for metastatic disease.

Despite the significant potential for tissue-based or serological biomarkers to help diagnose early stage disease, tailor therapy, or detect recurrence, very few biomarkers are in clinical use. Several tissue markers are used to help distinguish melanoma from other types of cancers, including S100, MART-1, and gp100/HMB45. To date, however, there are no tissue-based biomarkers that are utilized clinically for prognostic classification. This is despite the identification of multiple biomarkers, through genomic or immunohistochemical analysis, whose abnormal expression has been linked to poor outcome. These include tumor suppressors/oncogenes/signal transducers (p16, PTEN, EGFR, c-KIT, c-myc, bcl-6, HER3), cell-cycle associated proteins (Ki67, Cyclins A, B, D, E, p21, Geminin, PCNA), regulators of apoptosis (bcl-2, bax, Bak, ING3, ING4), proteins involved with cell adhesion and motility (P, E and N-cadherin, *β*-catenin, *β*1 and *β*3 integrins, matrix metalloproteinases (MMPs)), and others (Hsp90, RGS1, NCOA3, MCM4, MCM6) [3, 4].

There have also been several serologic markers that have been associated with poor prognosis. These include differentiation antigens (S100*β*, MIA, tyrosinase), proangiogenic factors (vascular endothelial growth factor (VEGF), basic fibroblast growth factor (BFGF), IL-8), cell adhesion and motility molecules (soluble intracellular adhesion molecule 1 (sICAM-1), soluble vascular cell adhesion molecule 1 (sVCAM), MMP-1, MMP-9), cytokines (IL-6, IL-10, soluble IL-2 receptor (sIL-2R)), and others (TA90 immune complex, YKL-40) [3–5]. However, only two of these are utilized clinically. 

The strongest prognostic serum biomarker is lactate dehydrogenase (LDH), which correlates with tumor load in advanced disease and is the strongest independent prognostic factor in stage IV melanoma [6]. It is the only biomarker included in the AJCC staging system [7] and is often used to stratify patients for randomized trials in stage IV disease. There is limited benefit, however, to the measurement of LDH among patients with earlier stage disease, particularly in the followup of patients who appear tumor-free after surgical resection.

The S100 protein consists of two subunits, alpha and beta. The beta subunit is expressed in cells of melanocytic lineage and is often used as an immunohistochemical marker for histological diagnosis of melanoma. Serum S100*β* has also been studied as a prognostic biomarker. Several studies have demonstrated an association between serum S100*β* levels and outcome, independent of stage. S100*β* serum concentrations can be a useful marker for monitoring therapy response in patients with advanced disease. While some European guidelines recommend routine S100*β* measurements as part of the surveillance of melanoma patients, [8, 9], there is limited evidence that this impacts outcome [10, 11].

## 3. Tissue Proteomics in Melanoma

Proteomic approaches can be divided into two categories: those that characterize the entire protein complement of the cells or tissue of interest and those that examine only those proteins found in specific specimens (primarily blood, but this could also include other fluids such as saliva or urine). Several groups have utilized functional proteomics to identify alterations in protein expression and posttranslational modifications to identify markers of melanoma progression as well as predictive markers, such as identifying proteins that may be associated with response to therapy (Table 1). While this approach has been utilized across a spectrum of primary tumors, it is more difficult in melanoma secondary to the limited accessibility of primary melanoma tissues. Therefore most of this work has been carried out in melanoma cell lines. 

Two-dimensional electrophoresis (2DE) has been the mainstay tool for separating proteins for many years. Proteins in a 2-dimensional gel are separated in the first dimension based on isoelectric points and then in a second dimension based on molecular masses. Differences between the samples can be compared and relative quantities determined by quantifying the ratios of spot intensities in the 2D gels. Matrix-assisted desorption/ionization time of flight mass spectrometry (MALDI-TOF MS) can then be used to analyze small amounts of protein isolated from the gel. The mass information obtained can then be used for protein identification using an appropriate protein sequence database and search program. 

Bernard et al. [12] used 2DE and mass spectrometry to identify proteins that differentiated melanocytes from melanoma cell lines and therefore may be important in the early progression to melanoma. Two proteins, nuclophosmin/B23 and hepatoma-derived growth factor (HDGF), were strongly upregulated in melanoma, while cathepsin D was downregulated in melanoma cell lines. Carta et al. [13] also used 2DE and mass spectrometry to examine the proteomes of cultured melanocytes and melanoma cell lines from primary and metastatic lesions. They identified several candidate proteins, many of which were stress proteins. They then used RT-PCR to evaluate mRNA expression of these proteins and found that overexpression of HSP27, HSP60, and HSPA8 and downregulation of PRDX2 were observed more commonly in metastatic melanoma versus primary melanoma.

As opposed to identifying proteins associated with melanoma development and progression, Sinha et al. [14] compared melanoma cell lines with varying degrees of resistance to commonly used anticancer drugs to identify proteins that may be responsible for resistance to therapy. Starting with a single melanoma cell line, they created a panel of sublines that exhibited different levels of drug sensitivity [15]. Using 2DE and matrix-assisted laser desorption/ionization-time of flight (MALDI-TOF) mass spectrometry for protein identification, they identified a variety of proteins that were differentially expressed in chemoresistant melanoma cell lines, many of which were chaperones, including heat-shock proteins (HSPs). 

These studies utilized electrophoresis to physically separate the proteins, requiring the use of melanoma cell lines. Hardesty et al. [16] used MALDI-imaging mass spectrometry (MALDI-IMS) analysis, which acquires information from intact proteins directly from thin sections of the tissue [17]. This allows for the analysis of specific cellular regions and precludes the need to generate a cell line from which the proteins are isolated. Using lymph nodes involved with metastatic melanoma from 69 stage III patients, they identified two proteins that were associated with recurrence, cytochrome C and calmodulin, with a better prognosis as the intensity of both proteins increase. 

While this approach is applicable to patients with larger metastatic deposits, such as clinically involved lymph nodes, it is not useful for discovery of primary tumor biomarkers. For approximately 90% of melanoma patients, the entire primary tumor is excised during the initial biopsy, so proteomic investigations requiring fresh or frozen primary tumor tissue are not applicable. Many academic centers have collections of formalin-fixed paraffin-embedded (FFPE) melanoma specimens, with accompanying clinicopathologic and outcome data. If these specimens could be used to scrutinize the proteome, the use of proteomics for melanoma biomarker discovery would take a giant leap forward. Unfortunately, FFPE tissues are typically refractory to proteomic investigations using today's methodologies, largely due to the high level of covalently linked proteins arising from formalin fixation [18]. Shotgun proteomics involves direct digestion of protein mixtures to complex peptide mixtures which are then separated and analyzed. These approaches can be used to extract proteins or peptides from fixed tissues for analysis. Several proteomics studies using FFPE tissue have been reported [19–22]. A newer approach, a modified shotgun proteomic strategy, termed direct tissue proteomics (DTP), can extract and identify peptides and proteins directly from tissues using micro-reverse-phase (*μ*RP) LC-MS/MS and has been proposed for use in melanoma [18, 23]. While there are still several obstacles to overcome, DTP with efficient extraction of proteins from FFPE tissue could open up a new avenue of retrospective proteomics-based biomarker investigation in melanoma.

## 4. Serum Proteomics

As discussed, examining the entire proteome of the cell of interest requires either the generation of a cell line or adequate harvestable tissue, which inherently limits and biases the study population. This presents several obstacles to both validating the findings and ultimately using them clinically. Blood carries not only plasma-specific proteins but also multiple proteins derived from other tissues, including tumors. Many proteins are secreted, shed, or lost into the circulation, either directly by tumor cells or indirectly after destruction of the tumor cells. The development, validation, and use of serum tests hold several potential advantages over tests that require primary tumor tissue, particularly for melanoma. As an example, Takikawa et al. [24] compared the serum proteome between healthy volunteers and melanoma patients using NanoLC and MALDI-TOF-MS, and identified 9 proteins detectable in plasma from the melanoma patients but not healthy plasma. Ultimately they identified pro-platelet basic protein precursor (PPBP) as a protein whose level corresponded with outcome and may serve as a serological prognostic biomarker. 

Several investigators have utilized surface-enhanced laser desorption/ionization (SELDI) and protein chip technology to find serum protein patterns that may be associated with the presence or the stage of melanoma. While gene chips have allowed for the detection of thousands of genes from very small samples, the creation of protein chips has faced several obstacles. Compared to DNA, proteins are not as robust and tend to denature. Proteins are more difficult to attach to chip surfaces, and while PCR can be used to amplify DNA, there is no method of amplifying minute amounts of protein. The ProteinChip Biology System uses SELDI-TOF MS to retain proteins on a solid-phase chromatographic surface that are subsequently ionized and detected by TOF MS [25, 26]. The surface of the ProteinChip is designed to select proteins from extracts due to either chemical (anionic, cationic, hydrophobic, hydrophilic) or biochemical (antibody, receptor, DNA, enzyme) properties. This is a more user-friendly approach to proteomics; SELDI has several advantages over other technologies for high-throughput screening as it is rapid, of relatively low cost, and reproducible. It requires smaller amounts of sample than 2DE as protein profiles can be made from fewer cells. It is also readily adaptable to a diagnostic format.

This technology can be used to detect protein expression patterns and then compare these patterns between different population sets. These patterns could be used as a clinical diagnostic test. Caron et al. [27] used this technology to try and discriminate between serum samples from melanoma patients and healthy volunteers and demonstrated a good diagnostic accuracy of 98.1% (sensitivity 96.7%, specificity 100%). Wilson et al. [28] examined the serum from patients with AJCC stage I or II melanoma who recurred (*n* = 25) or did not (*n* = 24) using SELDI ProteinChip mass spectrometry (MS) and identified three protein expression pattern differences that could discriminate between the two. Mian et al. demonstrated the potential of this technology with artificial neural networks (ANNs) to discriminate between serum samples from 101 stage I melanoma patients and 104 stage IV melanoma patients, as well as from 28 stage III patients who recurred from 27 stage III patients who did not [29]. Further research, focusing on the highest peak, ultimately identified serum amyloid A (SAA) as a potential prognostic biomarker in melanoma [30]. 

There are some drawbacks to serum-based proteomics. The expression and release of proteins into the serum can be variable. As an example of this, Greco et al. [31] obtained serum from 50 patients undergoing biopsy for suspected melanoma. Using 2DE and MALDI-TOF-MS, they identified increases of transthyretin (TTR) and angiotensinogen (AGT) and decreased expression of vitamin D binding protein (DBP). The investigators also examined serum samples after surgical removal of the melanoma and found that these were no longer elevated 1 month after surgery. This is an important consideration in melanoma patients, particularly if one is seeking to develop a prognostic serum test. This test would most likely be ordered after the diagnosis of melanoma and therefore might be greatly impacted by whether or not the entire tumor was removed with the diagnostic biopsy.

Another drawback to serum proteomics is that most (97%) of the proteins found in plasma belong to one of 7 major groups of high-abundance plasma proteins: albumin, immunoglobulins, fibrinogen, alpha-1 antitrypsin, alpha-2 macroglobulin, transferring, and lipoproteins. As these are primarily proinflammatory proteins, they are unlikely to represent prognostic or predictive biomarkers. SELDI pattern recognition studies do not depend on detecting low-abundance proteins, but depend instead on fluctuations of protein expression patterns. Therefore this technology, as well as others, is limited in specifically identifying low-abundance proteins that may be useful biomarkers.

## 5. Antibody-Based Proteomics

An alternate approach to examining the proteome directly is to screen for antibodies in the serum of patients with melanoma. As with serum-based proteomics, primary tumor tissue from each patient is not required, a significant advantage in melanoma patients. Another advantage is that antibodies are more sensitive and stable than proteins, which is clinically important in creating clinically useful diagnostic or prognostic tests. Antibody-based proteomics is particularly well suited to melanoma as the presence of an immune response to melanoma-associated antigens has been well documented [32–35]. Monitoring the presence or absence of antibodies in the serum that recognize specific tumor antigens can provide insights into the propensity of melanoma to metastasize, serving as biomarkers of melanoma biology and perhaps identifying ideal targets for therapeutic intervention. 

There are several high-throughput methods for the discovery of autoantibodies including serological screening of cDNA expression libraries (SEREX), phage display libraries and proteomics-based techniques. Serological protein analysis (SERPA) uses 2DE to separate proteins from tumor tissues or cell lines which are then transferred onto membranes by electroblotting and subsequently probed with sera from different populations of interest. Suzuki et al. [36] used this approach to identify 5 proteins that differentiated serum samples from melanoma patients and healthy volunteers (eukaryotic elongation factor 2 (EEF2), enolase 1 (ENO1), aldolase A (ALDOA), glyceraldehyde-3-phosphate dehydrogenase (GAPDH), and heterogeneous nuclear ribonucleoprotein (HNRNP-A2B1). 

Another proteomics-based approach to detecting serum autoantibodies is the use of protein microarrays. Protein microarrays spot proteins (purified, recombinant, or extracted from tumor cell lysates) onto microarrays which can be two-dimensional (glass slides, nitrocellulose membranes and microtitre plates) or three-dimensional (beads, nanoparticles). These are then incubated with sera from different populations. Advantages include the fact that less sample and reagents are needed, and autoantibodies to proteins with posttranslational modifications, such as glycosylated proteins, can be detected. There are, however, challenges to identifying the specific immune-reactive proteins in the respective protein fractions. Rather than screening the entire proteome, Liu et al. [37] focused on the subproteome of glycoproteins. Dual-lectin (ConA and WGA) affinity chromatography was applied to extract both glycoproteins from the lysate of a cell line generated from an intra-abdominal melanoma metastasis. Liquid-based reverse phase separation and natural protein microarray were then applied to separate the enriched proteins and spot the separated fractions on nitrocellulose slides. These were used to probe the sera from patients with newly diagnosed melanoma for antibodies that correlated with the presence of regional metastases. After validation, antibodies to 4 proteins including 94 kD glucose-regulated protein (GRP94), acid ceramidase (ASAH1), cathepsin D (CTSD), and lactate dehydrogenase B (LDHB) were identified that differentiated node-negative from node-positive patients.

## 6. Clinical Translation and Obstacles

Although the clinical potential of both tissue and serum-based proteomics in melanoma biomarker discovery seem strong, progress has been relatively limited to date. The reasons for this are multifactorial, both related to the inherent obstacles specific to melanoma and problems with study design. For the field to advance, several issues need to be tackled.

A key obstacle to biomarker discovery is reproducibility. This has been a significant problem with proteomics. As an example, in a meta-analysis of prostate cancer proteomic data obtained with SELDI-TOF, published results seem to differ greatly between different groups, and even from within the same groups [38, 39]. As evidenced in Table 1, there is little overlap between the proteins identified by different groups using different techniques. There are multiple approaches to proteomics, each with significant advantages and disadvantages depending on what the question is. Compared with serum proteomics, tissue proteomics has a higher likelihood of identifying marker candidates based on the higher concentration of protein within the tissue than after dilution in peripheral blood. For melanoma, the use of cell lines is most feasible however results are immediately biased by (1) selecting patients with harvestable tumor, (2) selecting melanomas that grow well *in vitro,* and (3) by changes in protein expression induced by *in vitro* culturing. Variations in cell culture technique could yield different results from the same cell line. Using tissue obtained directly from patients avoids some of these issues, but limits the patient population and hence the questions that can be asked. In addition, the results obtained can be greatly impacted by stroma, necrotic tissue, serum proteins, and blood cells within the specimen. Pure cancer cell populations can be created using fine needle aspiration, calcium starvation, immunomagnetic separation, or laser capture microdissection (LCM), [40–43] but this is extremely difficult to do when there is a limited supply of primary tumor tissue. Until newer technologies for examining FFPE tissues are more fully developed, this approach will be limited to select patient populations. Serum proteomics may identify fewer candidate proteins, but given the drawbacks of tissue proteomics in melanoma, this approach may be more translatable to clinical use. Examining the serum proteome is impossible without reducing the complexity of the protein mixture by removing highly abundant serum proteins, for which there are also several methods. When the blood is drawn, how it is stored, and whether serum or plasma is used can potentially impact the results. Therefore one can see that before any analysis is even performed, variations in the methods used to prepare the samples (cell culturing, tissue procurement, extraction of highly abundant serum proteins) can significantly impact the results. The methods used to do this must be highly reproducible, as even small variations in buffers or agents can alter the results. It becomes easy to see how radically different results can be reached even when the same experimental technique is utilized [44]. 

Quite often, initial experiments are designed based on the available samples and technologies, without as much forethought into the clinical question that the findings hope to address. In melanoma, for example, tissue proteomics are often carried out on samples from resected lymph nodes and metastatic deposits, as there is ample tissue available. Clinically, however, this is a population with a very poor prognosis, and outside of very specific questions, it is less likely that biomarkers identified from these highly dedifferentiated samples would impact clinical decision making or serve as useful biomarkers in patients with early stage disease. It is imperative that translational oncology be hypothesis driven, whereby even discovery studies are designed with a specific clinical question in mind. Even something as simple as when blood samples are obtained, if they do not reflect when they would be drawn in the clinical setting, could negatively impact the results. Close collaboration between clinical experts in melanoma and basic scientists is imperative to results that have a high likelihood of clinical impact.

Patient selection is also critical to results that are both reproducible and clinically relevant. In oncology, it is quite common to discover a biomarker among a highly heterogenous group of patients that on univariate analysis is significantly associated with outcome, but in reality correlates so strongly with known staging factors (tumor size, grade) that on multivariate analysis it provides no independent prognostic information. While these may be of scientific interest in unraveling the genes/proteins associated with dedifferentiation and metastases, they are of limited clinical benefit in stratifying patients beyond our current staging systems. It is therefore imperative that samples be obtained from as homogenous a population as is feasible and in adequate numbers so that newly discovered biomarkers are analyzed in the context of the known prognostic and predictive factors used in clinical decision making. Often smaller and more heterogeneous sample sets are chosen for practical reasons, which unfortunately contribute to the large number of reported biomarkers that are never validated or demonstrate clinical utility. 

Equally important is the appropriate selection of controls. For prognostic markers meant to differentiate between melanoma patients with different outcomes, it is imperative that the “good” cohort have adequate followup to be sure that this is truly a group at low likelihood of recurrence. Frequently these controls only have a median followup of 2-3 years. With longer followup, several of the “good” players may recur, and with small sample sets the conversion of only 1 or 2 patients from “good” to “bad” can dramatically change the results. Likewise, the search for biomarkers associated with melanoma development, with potential use as a screening tool, often compares melanoma patients (of varying stages) to healthy volunteers. Neither of these populations is appropriate—the melanoma group should only include patients with early stage, recently diagnosed disease, and the control group should not be healthy volunteers but rather high-risk individuals with similar characteristics regarding age and sun exposure. 

While the field of proteomics and other “-omics” fields are replete with articles describing discovery, there are dramatically fewer articles validating previously published results. Several reports include internal validations, using a fraction of their samples for discovery and then validating the results in the complete set. However, the bioinformatics tools used in discovery sets often seek to overfit the data, erring on the side of not missing a potential biomarker, but resulting in sensitivities and specificities that may not be reproducible. Even if an institution is able to validate their own findings, given the impact that sample preparation and patient selection can have, validation from other institutions is absolutely critical. Beyond interlaboratory variations, many biomarkers demonstrate varying expression based on patient characteristics (age, race and ethnicity, genetic lineage, environmental exposures). Therefore, biomarkers discovered and validated on a population in one geographic area may not be validated on another, even though the techniques are the same and the populations appear matched by known prognostic factors (Breslow thickness, nodal status, etc.). Unfortunately, there is limited enthusiasm for one institution to attempt and validate a published result. Validation requires larger numbers and can be costly and labor intensive. As many candidate biomarkers will not be validated, few researchers are interested in devoting time and money to a project that will likely not result in a publication, as there is limited interest on the part of prominent journals in publishing negative results. This leads to significant publication bias, which all of us as editors and reviewers are in part responsible for.

As new proteomic-based biomarkers are discovered, it is increasingly important that a mechanism exists by which the most promising biomarkers can be validated using external samples. This will require a collaborative effort on the part of the leading melanoma research centers. As an example of this, the National Cancer Institute has created the Early Detection Research, which hopes to promote biomarker discovery, validation, and translation into clinical practice for biomarkers associated with screening and risk [45]. Similar disease-specific collaborative efforts, centered on prospectively collecting data, blood and tumor tissue from multiple centers *not* for biomarker discovery but rather for validation, will be necessary to validate prognostic and predictive biomarkers.

## Figures and Tables

**Table 1:** Proteomics for biomarker discovery in melanoma.

Author	Year	Specimens	Comparison	Methodology	Proteins of interest
Bernard et al. [12]	2003	Cell lines	Melanocyte versus melanoma (primary and metastatic)	2DE and mass spectrometry	Nucleophosmin/B23, HDGF, CTSD
Sinha et al. [14]	2003	Cell lines	Responsive to chemotherapy versus nonresponsive	2DE and MALDI-TOF	Multiple (25)
Wilson et al. [28]	2004	Serum	Stage I or II melanoma patients, recurrence versus none	SELDI-TOF	n/a (expression profiles)
Carta et al. [13]	2005	Cell lines	Primary versus metastatic melanoma	2DE and mass spectrometry	HSP27, HSP60, HSPA8, PRDX2
Mian et al. [29, 30]	2005	Serum	Stage I melanoma versus Stage IV melanoma	SELDI-TOF	n/a (expression profiles), further work identified SAA
Mian et al. [29, 30]	2005	Serum	Stage III melanoma patients, recurrence versus none	SELDI-TOF	
Takikawa et al. [24]	2009	Serum	Volunteers versus melanoma patients	Nano LC and MALDI-TOF	PPBP
Caron et al. [27]	2009	Serum	Volunteers versus melanoma patients	SELDI-TOF	n/a (expression profiles)
Greco et al. [31]	2009	Serum	Patients undergoing biopsy, melanoma versus not	2DE and MALDI-TOF	TTR, AGT, DBP
Paulitschke et al. [46]	2009	Secreted proteins from and cell lines and skin samples	Normal skin versus Melanoma	Nano LC and mass spectrometry	GPX5, periostin, stanniocalcini-1
Suzuki et al. [36]	2010	Serum	Volunteers versus melanoma patients	Autoantibody detection	EEF2, ENO1, ALDOA, GAPDH, HNRNP-A2B1
Hood et al. [47]	2010	Cell lines	Melanoma versus normal skin	Nano LC and mass spectrometry	Tenascin-C, fibronectin, ACN4, TSP-1
Liu et al. [37]	2010	Serum	Node negative versus node positive melanoma patients	Autoantibody detection	GRP94, ASAH1, CTSD, LDHB
Hardesty et al. [16]	2011	Surgically resected lymph nodes	Recurrence versus no recurrence	MALDI-IMS	Cytochrome C calmodulin

HDGF: hepatoma-derived growth factor, CTSD: cathespin D, PPBP: prop-platelet basic protein precursor, SAA: serum amyloid A, TTR: transthyretin, AGT: angiotensinogen, DBP: vitamin D binding protein, EEF2: eukaryotic elongation factor 2, ENO1: enolase 1, ALDOA: aldolase A, GAPDH: glyceraldehyde-3-phosphate dehydrogenase, HNRNP-A2B1: heterogeneous nuclear ribonucleoprotein A2B1, ACN4: alpha-actinin-4, TSP-1: thrombospondin-1, GRP94: 94 kD glucose-regulated protein, ASAH1: acid ceramidase, LDHB: lactate dehydrogenase B.
